# The SNAIL/miR-128 axis regulated growth, invasion, metastasis, and epithelial-to-mesenchymal transition of gastric cancer

**DOI:** 10.18632/oncotarget.16849

**Published:** 2017-04-05

**Authors:** Wei-Wei Yu, Hong Jiang, Cun-Tai Zhang, Yang Peng

**Affiliations:** ^1^ Department of Geriatrics, Tongji Hospital, Tongji Medical College, Huazhong University of Science and Technology, Wuhan 430030, China

**Keywords:** gastric cancer, miR-128, SNAIL, DNA methylation, transcription factor

## Abstract

miR-128 is expressed in various tumors, but its expression and function in gastric cancer have not been defined. Thus, the goal of this study was to characterize miR-128 in gastric cancer. We found first that miR-128 is down-regulated in gastric cancer cell lines and tissues, and this dysregulation is correlated with DNA methylation and the transcription factor SNAIL. Using prediction tools, western blotting, and luciferase reporter assays, we found that Bmi-1 was the direct target of miR-128. Additionally, overexpression of miR-128 inhibited gastric cancer cell migration, invasion, and proliferation by targeting Bmi-1 *in vitro* and *in vivo*. We also documented, with receiver operating characteristic curves and Kaplan-Meier survival analysis, that miR-128 and Bmi-1 may be useful markers for diagnosing and estimating the prognosis of gastric cancer patients. As the epithelial-to-mesenchymal transition is an important mechanism associated with cancer invasion and metastasis, we inferred that miR-128 could regulate this mechanism in gastric cancer. In fact, we found that miR-128 could reverse epithelial-to-mesenchymal transition induced by Bmi-1 via the PI3K/AKT pathway. Because SNAIL also acts as a mesenchymal marker, our findings identified a novel positive feedback loop in which the transcription factor SNAIL curbs the expression of miR-128, and then down-regulated miR-128 promotes the expression of Bmi-1; finally, overexpression of Bmi-1 drives the epithelial-to-mesenchymal transition process via the PI3K/AKT pathway, and the expression of SNAIL is up-regulated.

## INTRODUCTION

Gastric cancer is one of the most common cancers in the world [1]. In 2008, approximately 989,000 new cases (7.8% of global cancer totals) and 738,000 deaths (9.7% of global cancer totals) were recorded, making gastric cancer the fourth most common malignancy and the second leading cause of cancer death worldwide [2]. The high death rate is due to the propensity for gastric cancer to metastasize and invade widely. Many factors are implicated in the development of gastric cancer, including Helicobacter pylori infection, unhealthy diet, and smoking [3–5]. The genesis and progression of human gastric cancer are thought to be also influenced by genetic and epigenetic alterations, including the activation of oncogenes and the inactivation of tumor suppressor genes.

Published evidence [6] indicates that both oncogenes and tumor suppressor genes can be regulated by microRNAs (miRNAs). miRNAs are non-coding RNA molecules, approximately 21-23 nucleotides in length, which regulate gene expression at the post-transcriptional level [7–9]. It has been reported [10, 11] that miRNAs can regulate many malignant phenotypes of cancer, such as cancer cell proliferation, apoptosis, migration, and invasion. Thus, miRNAs can function as tumor suppressors or oncogenes, and deregulated miRNA expression might contribute to tumor cell metastasis.

miR-128 is expressed differently in various tumors. Some studies show that miR-128 acts as a tumor suppressor. For example, Evangelisti et al. [12] found that up-regulation of miR-128 reduces neuroblastoma cell motility and invasiveness; Khanet et al. [13] found that miR-128 can curb the proliferation and invasion of prostatic cancer cells; and Hu et al. [14] found that miR-128 inhibits tumorigenesis, angiogenesis, and lymphangiogenesis of human non-small cell lung cancer by directly targeting vascular endothelial growth factor-C. Conversely, other studies have shown that miR-128 acts as an oncogene. Thus, Mets et al. [15] found that miR-128 is a candidate oncogenic miRNA in T-cell acute lymphoblastic leukemia, which targets the PHF6 tumor suppressor gene, and Zhuang et al. [16] found that expression of miR-128 in hepatocellular carcinoma tissues was up-regulated compared with its expression in adjacent non-tumor tissues. However, the function and expression of miR-128 in gastric cancer are still undefined.

In this study, we examined the expressions of miR-128 in gastric cancer and the diagnostic and prognostic potential of miR-128 in this cancer. To investigate why miR-128 is dysregulated in gastric cancer, we studied the up-stream genes and the methylation of miR-128. Finally, we investigated the role of miR-128 and its down-stream gene in growth, invasion, and metastasis of gastric cancer *in vitro* and *in vivo*.

## RESULTS

### miR-128 is down-regulated in gastric cancer cell lines and tissues

**Figure 1:**
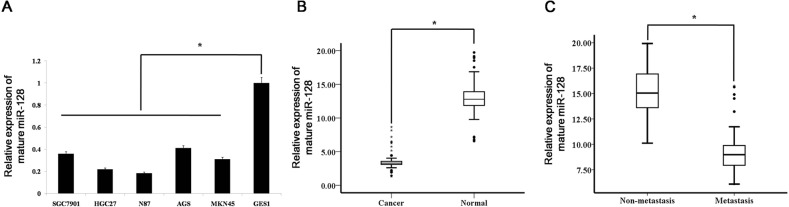
miR-128 is down-regulated in gastric cancer tissues and cell lines **(A)** miR-128 expression was detected in gastric cancer cell lines (SGC7901, HGC27, AGS, MKN45, and NCI-N87) and a normal gastric mucosa cell line, GES1. Data are shown as the mean ± s.d. (n = 3) in cell lines; * p < 0.05. **(B)** The expression level of mature miR-128 in gastric cancer (n = 135) or adjacent normal mucosa tissues (n = 135) was determined by qRT-PCR analysis. Data are shown separately in human samples; * p<0.05. **(C)** Mature miR-128 expression levels in metastatic (n = 83) and non-metastatic (n = 52) gastric cancers. Data are shown separately in human samples; * p < 0.05.

To investigate the miR-128 expression levels in human gastric cancer cell lines, we monitored its expression in several cell lines (SGC7901, HGC27, AGS, MKN45 and NCI-N87) and in a normal gastric mucosa cell line (GES1). We found that expression of miR-128 was reduced in the gastric cancer cell lines compared with normal gastric mucosa cells (Figure 1A).

To further explore the roles of miR-128 in human gastric cancer, we measured the levels of miR-128 expression in 135 human gastric cancer samples and 135 adjacent normal mucosa samples. Clinical information for the patients is shown in Table 1. According to qRT-PCR analysis, the levels of miR-128 expression were significantly decreased in tumor tissues compared to levels in the adjacent normal mucosa (Figure 1B). To determine whether miR-128 expression is associated with gastric cancer metastasis, we examined miR-128 expression levels in 135 archived primary gastric tumors, divided into two groups: the tumors in one group had been resected from 83 patients with lymph node metastases, whereas the tumors in the other group had been resected from 52 patients without metastases. According to the qRT-PCR analysis, the miR-128 expression levels were significantly lower in tumors from the patients with metastases than from tumors in the patients without metastases (Figure 1C).

**Table 1:** Clinicopathological characteristics of the patient cohort

Characteristics	Total cases (n = 135)
Age (year)	
Range	33-88
Mean	54
Median	57
Pathological T	
PT_0_-T_is_	33(24.4%)
PT_1_	28(20.7%)
PT_2_	24(17.8%)
PT_3_	18(13.4%)
PT_4_	32(23.7%)
Lymph node metastases	
PN_0_	52(38.5%)
PN_1-3_	83(61.5%)
Dead/alive	
Dead	85(63.0%)
Alive	19(14.1%)
Missing	31(22.9%)

### miR-128 is epigenetically silenced in gastric cancer

**Figure 2:**
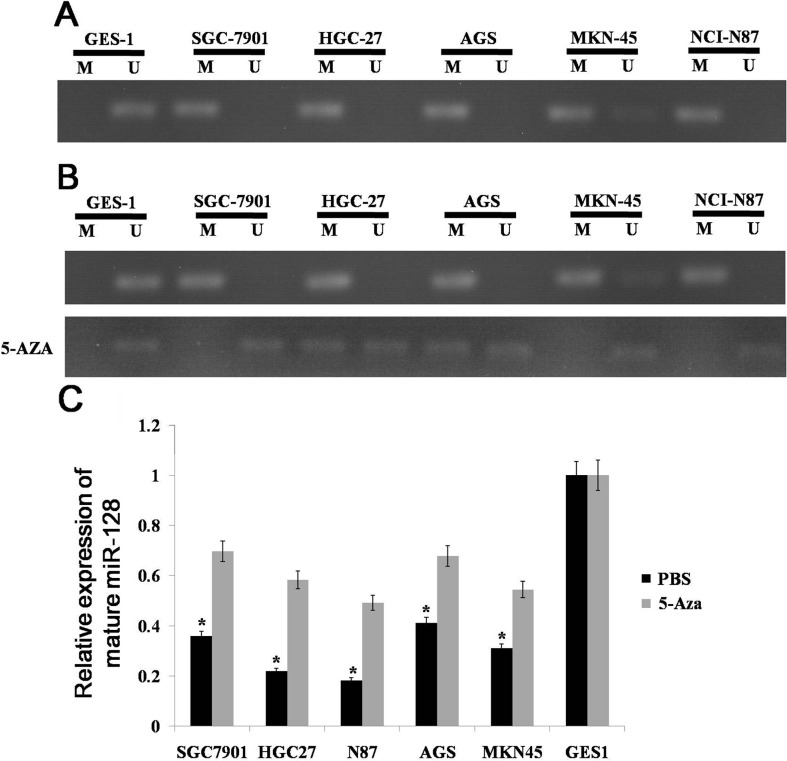
Methylation status of miR-128 CpG island **(A)** miR-128 methylation was detected by MSP analysis in gastric cancer cell lines. **(B)** miR-128 methylation was detected by MSP analysis with or without 5-aza-dCyd treatment in gastric cancer cell lines. M: MSP of methylation-specific primers; U: MSP of non-methylation-specific primers. **(C)** miR-128 expression was examined by qRT-PCR in gastric cancer cell lines and a normal gastric mucosa cell line. Data are shown as the mean ± s.d. (n = 3) in cell lines; * p < 0.05.

We know that miR-128 is down-regulated in gastric cancer, but the cause of the down-regulation is unknown. As epigenetic silencing is a common means of down-regulating miRNA expression [17, 18], we made an assumptionthat miR-128 can be silenced by DNA methylation. To examine this possibility, we first analyzed the CpG island distribution in the promoter region of miR-128 by the use of Methyl Primer Express v1.0 software. We found that CpG islands exist in the upstream of the transcription start site. We then investigated the methylation status of CpG islands in the promoter region with methylation-specific PCR (MSP); we found that the miR-128 CpG island was extensively methylated in gastric cancer cell lines compared to the methylation status in GES-1 cells (Figure 2A). To further determine whether the expression of miR-128 was silenced by DNA methylation, we determined miR-128 expression in gastric cancer lines with or without 5-aza-dCyd treatment. We found that the miR-128 CpG island was demethylated in gastric cancer lines treated with 5-aza-dCyd but not in those treated with control phosphate-buffered saline (Figure 2B). Additionally, miR-128 expression was significantly increased in the cell lines treated with 5-aza-dCyd but not in control cell lines (Figure 2C). From these results, we conclude that miR-128 is epigenetically silenced in gastric cancer.

### miR-128 is directly repressed by the transcription factor SNAIL

**Figure 3:**
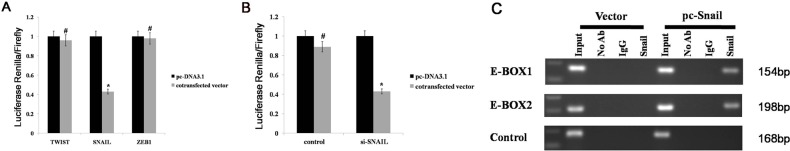
miR-128 is directly repressed by SNAIL **(A)** The SNAIL, TWIST, and ZEB1 expressing vector was co-transfected with various constructs containing the 3-kb fragment upstream of human miR-128-2 stem-loop into AGS cells. **(B)** si-SNAIL was co-transfected with miR-128 stem-loop plasmid into AGS cell lines, and luciferase activity was recorded. **(C)** ChIP assay analysis was performed in AGS cells transfected with a vector expressing SNAIL. Data are shown as the mean ± s.d. (n = 3) in cell lines; * p < 0.05.

We have found that miR-128 can be regulated by DNA methylation, but it is not known whether any transcription factors can regulate miR-128 expression in gastric cancer. Two conserved E-box motifs relative to the transcription start site of the human pri-miR-128 stem-loop have been identified [19] and Peinado et al. [20] found that SNAIL, ZEB, and the bHLH families can bind to E-box sequences in the promoters. In this study, to detect which transcription factors can regulate miR-128 expression, we constructed TWIST (bHLH family), SNAIL (SNAIL family), and ZEB1 (ZEB family) expression vectors, and separately co-transfected the vectors with other constructs containing a 3-kb fragment upstream of the human miR-128 stem-loop into AGS cells. We found that the relative luciferase activity of miR-128 was significantly decreased by SNAIL but not by ZEB1 or TWIST1 (Figure 3A). Conversely, when we co-transfected si-SNAIL with the miR-128 stem-loop plasmid into AGS cell lines, the luciferase activity of miR-128 was significantly increased (Figure 3B). To determine whether SNAIL can directly regulate miR-128, ChIP assays were performed. These assays revealed that SNAIL bound to both E-box-1 and E-box 2 (Figure 3C). This result is evidence that SNAIL can directly curb miR-128 expression by binding to E-box 1 and E-box 2, which are present in the promoters of the human pri-miR-128 stem-loop.

### miR-128 directly targets Bmi-1

**Figure 4:**
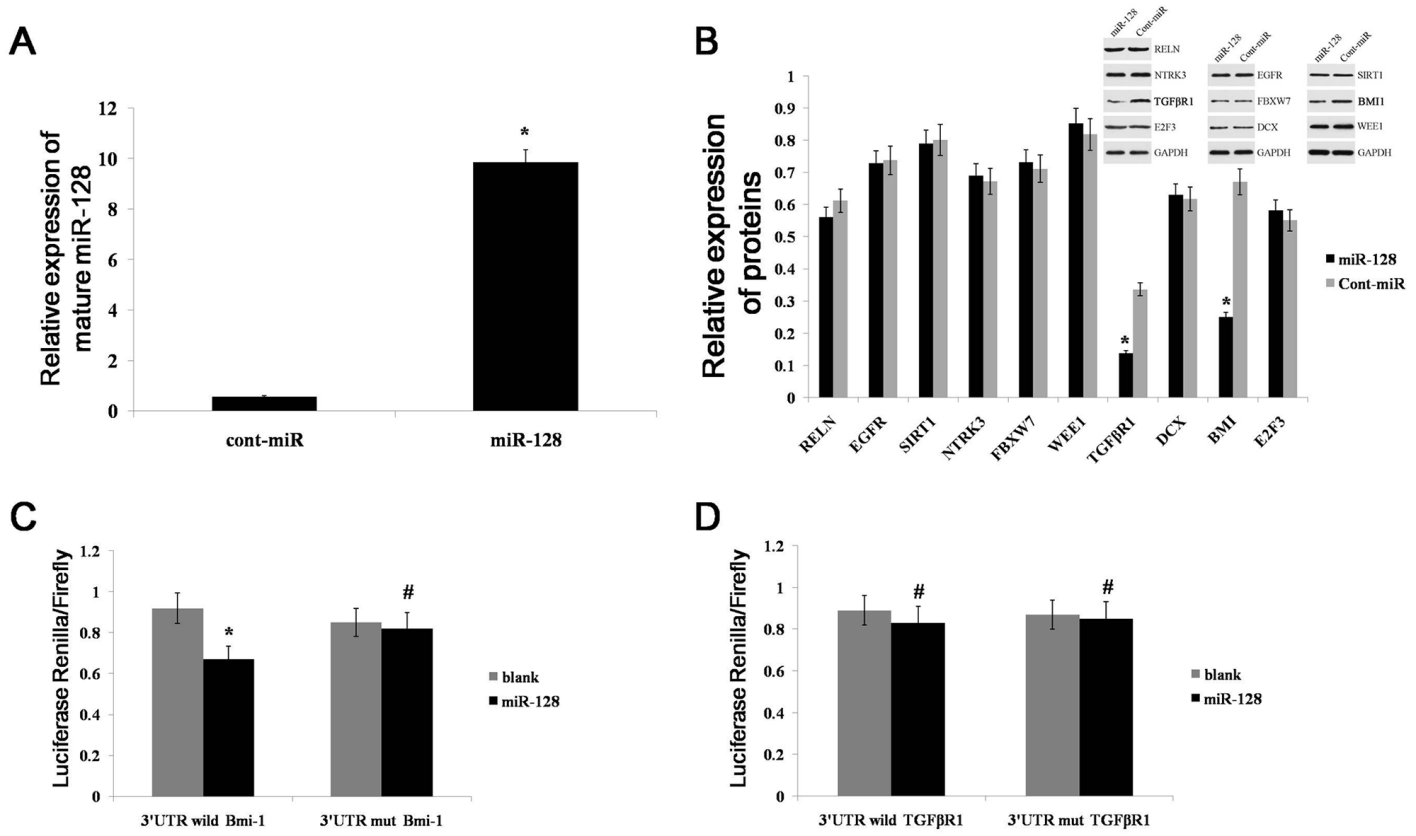
miR-128 can directly target Bmi-1 expression **(A)** miR-128 expression was detected in the AGS gastric cell line after transfection with miR-128 mimics or cont-miR. **(B)** Expression of 10 proteins was examined with western blot in the AGS gastric cell line after transfection with miR-128 mimics or cont-miR. **(C)** The relative luciferase activity was analyzed after the Bmi-1 reporter plasmids were co-transfected with miR-128 mimics or control mimics into AGS cell lines. **(D)** The relative luciferase activity was analyzed after the TGFβR1 reporter plasmids were co-transfected with miR-128 mimics or control mimics into AGS cell lines. Data are shown as the mean ± s.d. (n = 3) in cell lines; * p < 0.05, # p > 0.05.

By using the prediction tools Miranda (http://www.microrna.org), PicTar (http://www.pictar.org/), TargetScan (http://genes.mit.edu/targetscan/index.html), and miRDB (mirdb.org/miRDB/), we predicted the presence of 308 target genes of miR-128. Of those genes, we found that approximately 10 are likely down-stream target genes of miR-128, as they were identified by persuasive evidence (reporter assay, western blot, or qRT-PCR) using miRtarbase (http://mirtarbase.mbc.nctu.edu.tw/) (Table 2). We detected expression of these 10 genes in the AGS gastric cell line by western blot after transfecting cells with miR-128 mimics. Compared with the expression of cont-miR, miR-128 expression was significantly increased in the AGS cell line after transfecting cells with miR-128 mimics (Figure 4A). We also found that Bmi-1(B cell-specific Moloney murine leukemia virus integration site 1) and TGFβR1 expression were significantly decreased in miR-128 high-expressing gastric cancer cell lines, whereas the expression of other genes was unchanged (Figure 4B). Bmi-1 is a transcriptional repressor belonging to the polycomb group and is associated with cell proliferation and carcinogenesis in a variety of human cancers [21–24]. TGFβR1 is also closely associated with various tumors. We next performed luciferase reporter assays to determine whether miR-128 directly targets Bmi-1 and TGFβR1. We found that the co-transfection of miR-128 and the wild-type BMI 3′UTR caused a significant decrease in luciferase expression compared with the expression in controls, whereas co-transfection of miR-128 and the mutant Bmi-1 3′UTR did not (Figure 4C). Interestingly, co-transfection with miR-128 and the wild-type TGFβR1 3′UTR did not cause a significant decrease in luciferase expression (Figure 4D). Thus, it appears that miR-128 decreased the expression of TGFβR1 in an indirect way, whereas Bmi-1 is a direct target gene of miR-128.

**Table 2:** The probable down-stream target genes of miR-128

Gene	Strong evidence			Less strong evidence				
	Report assay	Western blot	qRT-PCR	Microarray	NGS	pSILAC	Other	Sum
**NTRK3**	√						√	**2**
**DCX**	√	√	√				√	**4**
**RELN**	√	√	√				√	**4**
**E2F3**	√						√	**2**
**EGFR**		√					√	**2**
**BMI1**	√	√	√	√			√	**5**
**TGFBR1**	√	√			√		√	**4**
**FBXW7**	√	√		√	√		√	**5**
**WEE1**	√		√	√				**3**
**SIRT1**	√							**1**

### miR-128 inhibits gastric cancer cell migration, invasion, and proliferation by targeting Bmi-1

**Figure 5:**
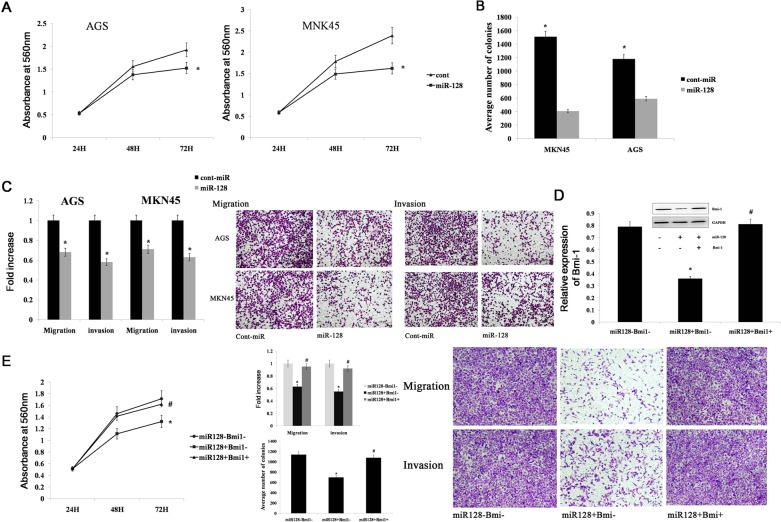
miR-128 inhibits gastric cancer migration, invasion, and proliferation by targeting Bmi-1 **(A)**, **(B)** Cell proliferation and the colony-forming ability was shown after transfection with miR-128 mimics or Cont-miR in gastric cancer cell lines AGS and MKN45. **(C)** The migration and invasion of AGS and MKN45 cell lines are illustrated after transfection with miR-128 mimics or Cont-miR. **(D)** Immunoblot analysis of Bmi-1 expression in AGS cells transfected with miR-128 mimics or cont-miR, with or without Bmi-1 restoration. **(E)** The tumourigenic qualities of AGS cells were assessed after transfection with miR-128 or cont-miR, with or without Bmi-1 restoration. Cell proliferation is illustrated in the left panel. Gastric cancer migration and invasion are illustrated in the right panel. Data are shown as the mean ± s.d. (n = 3) in cell lines; * p < 0.05, # p > 0.05.

To study the functional significance of miR-128 in gastric cancer, we transfected the AGS and MKN45 gastric cancer cell lines with miR-128 mimics. We found that the cells with forced expression of miR-128 had significantly decreased proliferation compared with the proliferation of the cells with forced expression of cont-miR (Figure 5A). The forced miR-128 cells also exhibited reduced ability to form colonies; the number of foci in the forced miR-128 cells was decreased compared with the numbers in cont-miR-treated cells (Figure 5B). Additionally, Transwell migration and Matrigel invasion assays demonstrated that miR-128 significantly decreased the migration and invasion of AGS and MKN45 cells (Figure 5C). To determine whether miR-128 promotes gastric cancer migration, invasion, and proliferation by targeting Bmi-1, we forced the expression of miR-128 in AGS cell lines together with a construct containing the Bmi-1 coding sequence but lacking the 3′UTR of Bmi-1 mRNA. This construct yielded a Bmi-1 mRNA that was resistant to miR-128. The restoration of Bmi-1 expression was confirmed by western blot experiments (Figure 5D). We found that gastric cancer cell migration, invasion, and proliferation were completely restored in the AGS cell line with forced miR-128 expression and Bmi-1 restoration (Figure 5E). Thus, we conclude that miR-128 inhibits gastric cancer cell migration, invasion, and proliferation by targeting Bmi-1.

### miR-128 induces reversion of epithelial-to-mesenchymal transition via Bmi-1 through the PI3K/AKT pathway

**Figure 6:**
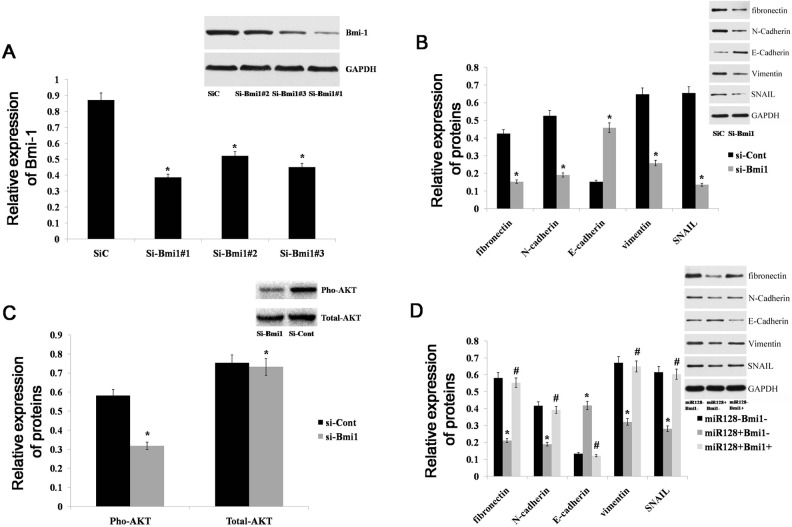
miR-128 promotes an epithelial phenotype in gastric cancer **(A)** Bmi-1 expression was detected with western blot in AGS cells after treatment with 3 independent siRNA sequences (si-Bmi1) or a control (si-Cont). **(B)** An immunoblot analysis of N-cadherin, vimentin, fibronectin, E-cadherin, and SNAIL in AGS cells transfected with si-Bmi1 or si-Cont. **(C)** Immunoblot analysis of pho-AKT and total-AKT in AGS cells transfected with si-Bmi1 or si-Cont. **(D)** Immunoblot analysis of N-cadherin, vimentin, fibronectin, E-cadherin, and SNAIL in AGS cells transfected with miR-128 mimics or cont-miR, with or without Bmi-1 restoration. Data are shown as the mean ± s.d. (n = 3) in cell lines; * p < 0.05.

Epithelial-to-mesenchymal transition (EMT) is characterized by loss of epithelial cell markers, such as E-cadherin, and gain of a mesenchymal phenotype expressingmesenchymal proteins, including N-cadherin, vimentin, fibronectin, and SNAIL [25]. EMT is an important mechanism in cancer invasion and metastasis. Many studies have shown that Bmi-1 can regulate EMT in various cancers [26–28], but it is unclear whether Bmi-1 can drive the EMT process in gastric cancer. To explore the role of Bmi-1 in establishing a mesenchymal phenotype in gastric cells, we deleted Bmi-1 using RNA interference (RNAi) (Figure 6A) and examined the expression of the mesenchymal markers fibronectin, vimentin, N-cadherin and SNAIL, as well as the epithelial marker E-cadherin, in AGS cells. We found that the expression levels of fibronectin, vimentin, N-cadherin, and SNAIL were decreased, but the expression of E-cadherin was increased in the Bmi-1- depleted tumor cells (Figure 6B). Genetic and cancer biology evidence has demonstrated that the PI3K/AKT pathway is a central mechanism controlling EMT [29–31]. Thus, we examined p-AKT expression in Bmi-depleted AGS cells and found that p-AKT expression was significantly decreased (Figure 6C). This result indicates that Bmi-1 can drive the EMT process by activating the PI3K/AKT pathway in gastric cancer. Because Bmi-1 is a direct target gene of miR-128, we inferred that miR-128 could determine the epithelial phenotype of gastric cancer by regulating Bmi-1. Accordingly, we examined the expression of mesenchymal and epithelial markers in miR-128-expressing AGS cells with or without Bmi-1 restoration. We found that the protein levels of fibronectin, vimentin, N-cadherin, and SNAIL were decreased in miR-128-expressing AGS cells, whereas the expression of E-cadherin was increased. Additionally, the expression of EMT markers in miR-128-expressing cells was restored to normal levels by the restoration of Bmi-1 expression (Figure 6D). Altogether, these results demonstrated that miR-128 could inhibit EMT via Bmi-1 through the PI3K/AKT pathway in gastric cancer cells.

### Diagnostic and prognostic significance of miR-128 and Bmi-1 in gastric cancer

**Figure 7:**
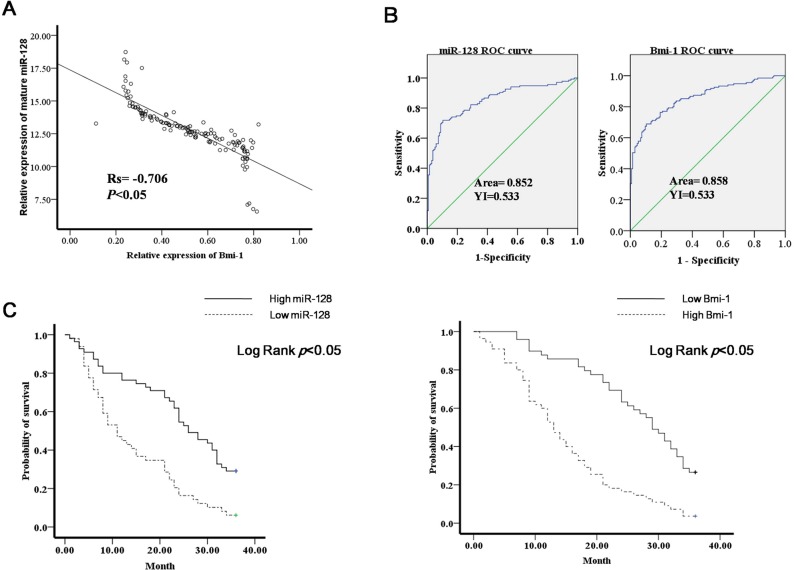
Diagnostic and prognostic significance of miR-130a in gastric cancer **(A)** Bmi-1 protein expression was examined with western blot in gastric cancer tissues; miR-128 expression and Bmi-1 protein expression were correlated; p < 0.05. **(B)** ROC curve analysis showing performance of miR-128 and Bmi-1 expression to discriminate between malignant and non-malignant tissue samples. **(C)** Kaplan-Meier analysis of patients' overall survival based on miR-128 and Bmi-1 expression.

From the results of the experiments described above, we knew that Bmi-1 is a direct target gene of miR-128. To further analyze the relationship between Bmi-1 and miR-128, we examined the expression of Bmi-1 in 135 gastric cancer tissues and adjacent normal mucosal tissues by western blot. We found that the expression of miR-128 was inversely correlated with that of Bmi-1 (Figure 7A). Receiver operating curve (ROC) analyses were performed to evaluate the ability of miR-128 and Bmi-1 expression to be used to discriminate between normal and tumor tissue samples. We found that miR-128 and Bmi-1 had the best sensitivity and specificity when the miR-128 Ct value was 5.87 (YI = 0.533), the Bmi-1 Ct value was 0.51(YI = 0.533), and an area under the reporter operator curve suggested that miR-128 and Bmi-1 can discriminate between malignant and non-malignant samples (Figure 7B). To determine whether the levels of miR-128 and Bmi-1 are correlated with the survival of gastric cancer patients, Kaplan-Meier survival analysis was performed. We divided the cases into low miR-128 and high miR-128 groups and low Bmi-1 and high Bmi-1 groups. The high miR-128 patients had significantly better overall survival than the low miR-128 patients. Conversely, the low Bmi-1 patients had significantly better overall survival than the high Bmi-1 patients (Figure 7C).

### miR-128 decreases tumor growth and metastasis by targeting Bmi-1 *in vivo*

**Figure 8:**
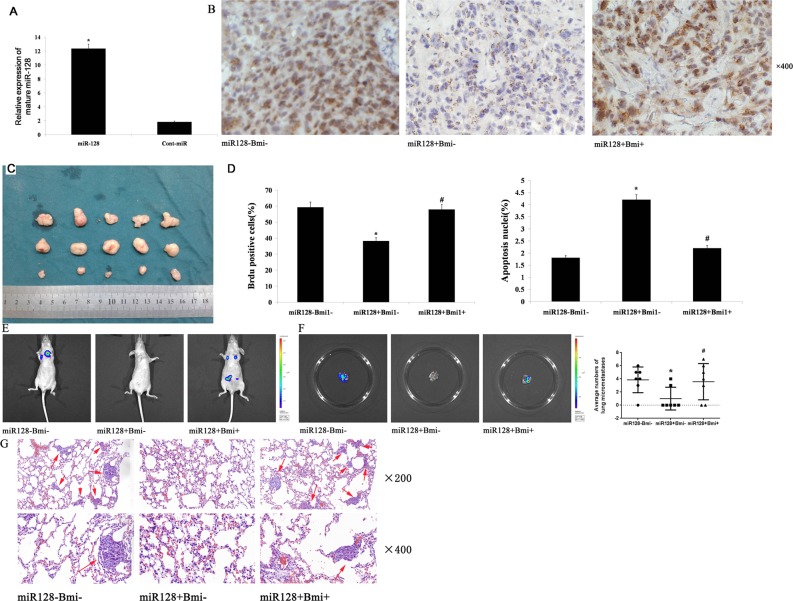
miR-128 decreases tumor growth and metastasis by Bmi-1 in nude mice **(A)** miR-128 expression in tumor xenografts was measured with qRT-PCR. **(B)** Bmi-1 expression in tumor xenografts was detected with immunohistochemical staining. **(C)** Images of xenograft tumors from nude mice with forced expression of miR-128 or cont-miR, with or without Bmi-1 restoration. **(D)** Evaluation of nuclear BrdU incorporation and TUNEL-positive (apoptotic) nuclei in xenograft tumors cells. **(E)** A representative IVIS imaging for metastasis in nude mice that had received tail-vein injection of miR-128 or cont-miR, with or without Bmi-1 infected AGS cells. **(F)** IVIS imaging for pulmonary metastasis foci and quantification for them pulmonary metastasis foci of three groups. **(G)** Hematoxylin and eosin-stained sections of lungs isolated from nude mice and pulmonary metastases detected in the lungs (red arrow). The data represent the means ± s.d.; * p < 0.05, # p > 0.05.

Based on the observed decreases in migratory, invasive, and proliferative behaviors in AGS and MKN45 cells transfected with miR-128, we next investigated the role of miR-128 in the growth of cells *in vivo*. We inoculated nude mice subcutaneously with equal numbers (1×10^6^ cells per mouse) of stable AGS cells with forced expression of miR-128 or cont-miR, with or without Bmi-1 restoration. miR-128 expression in the tumor xenografts was detected with qRT-PCR. We found that miR-128 expression was significantly increased in the tumors with overexpressed miR-128 (Figure 8A). Next, we examined Bmi-1 expression in tumor xenografts with immunohistochemistry. We found that Bmi-1 expression was significantly decreased in the tumors that overexpressed miR-128 and that Bmi-1 expression was restored in tumors with overexpressed Bmi-1 (Figure 8B). We also measured the volume of tumor xenografts and found that the forced expression of miR-128 significantly inhibited tumor growth *in vivo*, and overexpression of Bmi-1 could restore the tumor growth (Figure 8C). Furthermore, we found that forced expression of miR-128 correspondingly decreased cell proliferation, determined by immunohistochemical analysis of the nuclear incorporation of BrdU, and increased cell apoptosis, determined with a TUNEL assay. These effects could be reversed by overexpression of Bmi-1 (Figure 8D). We also determined the effect of miR-128 on metastasis *in vivo*. Accordingly, we injected stable AGS cells with forced expression of miR-128, with or without Bmi-1 restoration, into the venous circulation of mice. After 7 weeks, AGS cells with forced expression of miR-128 generated fewer lung metastases than did control cells, and the overexpression of Bmi-1 restored the propensity for tumor metastasis (Figure 8E–8G).

## DISCUSSION

Recent research has found that miR-128 is dysregulated in various cancers. In prostate cancer [32], neck squamous cell carcinoma [33], and lung cancer [14], miR-128 expression is down-regulated, whereas in osteosarcoma [34], hepatocellular carcinoma [16], and T-cell acute lymphoblastic leukemia [15], miR-128 expression is up-regulated. In gastric cancer, however, the function and role of miR-128 have been unclear. This knowledge deficit prompted us to critically characterize miR-128 in gastric cancer. We found that miR-128 expression was reduced in gastric cancer cell lines and tissues and that miR-128 expression was significantly lower in patients with metastases than in patients without metastases. Thus, it was evident that miR-128, a tumor suppressor gene, is down-regulated in gastric cancer and is associated with metastatic disease.

However, why the expression of miR-128 is down-regulated in gastric cancer remained to be answered. Recent studies have shown that DNA methylation can directly induce miRNA transcriptional repression. Shen et al. [35] reported that the expression of miR-125b and miR-199a is regulated by DNA hypermethylation in hepatocarcinogenesis, and Yang et al. [36] found that miR-129-2, which functions as a tumor suppressor in glioma cells, is down-regulated by DNA methylation. Thus, we inferred that miR-128 can be silenced by DNA methylation in gastric cancer. In this study, we predicted the presence of the miR-128 CpG island and examined the methylation status of the CpG island in the promoter region. We found that the miR-128 CpG island was extensively methylated in gastric cancer cells. Additionally, miR-128 expression was significantly increased when we demethylated the methylated DNA by 5-aza-dCyd treatment in gastric cancer cells, a result which indicated epigenetic silencing of miR-128. Interestingly, we found that miR-128 expression could not be completely restored by treatment with 5-aza-dCyd, a finding suggesting that miR-128 is regulated by an additional mechanism(s).

Some studies have demonstrated that miR-128 can be regulated by transcription factors. Donzelli et al. [37] found that mutant transcription factor p53 could control miR-128-2 expression at the transcriptional level, and others [38] have shown that SNAIL can negatively regulate miR-128. Tao et al. [39] found that SNAIL regulates miR-128 expression at the transcriptional level. We, therefore, inferred that, in gastric cancer, miR-128 also may be regulated by transcription factors. With a ChIP assay and luciferase reporter assay, we found that SNAIL can directly curb miR-128 expression by binding to E-box 1 and E-box 2, which are located in the transcription start site of the human pri-miR-128 stem-loop. SNAIL is a zinc finger transcriptional repressor, which consists of SNAI1 (SNAIL), SNAI2 (SLUG) and SNAI3 (SMUC). The targets of SNAI1 have been shown to be involved in tumor development [40] and tumor recurrence [41]. Some studies have shown that SNAIL can regulate proliferation, invasion, and apoptosis in various cancers by modulating miRNAs. Xu et al. [42] found that SNAIL-regulated miR-375 inhibits migration and invasion of gastric cancer cells by targeting JAK2. According to our results, miR-128 expression was down-regulated by methylation of the miR-128 CpG island DNA and SNAIL. Thus, we propose that low expression of miR-128 is associated with the occurrence and development of gastric cancer.

To further understand the function and role of miR-128, we used prediction tools and luciferase reporter assays to search for the direct downstream gene of miR-128 in gastric cancer cells, and we found the gene to be Bmi-1. Additionally, in gastric tissues, Spearman's rank test revealed that the expression of miR-128 was inversely correlated with that of Bmi-1. Bmi-1 was first isolated as an oncogene that cooperates with c-Myc in generating lymphomas in a murine model [43]. Bmi-1 protein is significantly overexpressed in ovarian, endometrial, and cervical cancer [44], and Sasaki et al. [45] found that overexpression of Bmi-1 and EZH2 is associated with the malignant progression of hepatocellular carcinoma. Bmi-1 was also found to be associated with gastric cancer. Wei et al. [46] found that Bmi-1 is critical for the proliferation and invasion of gastric carcinoma cells. Thus, we speculated that miR-128 regulates gastric cancer tumorigenesis by directly targeting Bmi-1. In fact, decisive experiments revealed that miR-128 can inhibit gastric cancer cell migration, invasion, and proliferation *in vitro*. Moreover, these changes were restored with overexpression of Bmi-1. In the orthotopic implantation model, overexpression of miR-128 significantly inhibited tumor growth, and the overexpression of Bmi-1 restored tumor growth. Furthermore, forced expression of miR-128 significantly decreased lung metastases in a metastatic nude mouse model, whereas overexpression of Bmi-1 restored the propensity for lung metastasis. Overall, our results show that miR-128 can inhibit gastric cancer migration, invasion, proliferation, and metastasis by targeting Bmi-1 *in vivo* and *in vitro*.

Some miRNAs have potential diagnostic value in various cancers [47, 48]. In our study, we found that miR-128 and Bmi-1 can be used as diagnostic markers for gastric cancer, and high levels of miR-128, as well as low levels of Bmi-1, were significantly associated with long overall survival of gastric cancer patients. Thus, miR-128 may serve as a molecular diagnostic and prognostic marker in gastric cancer patients.

Metastases are the primary cause of death from gastric cancer, and EMT is closely associated with gastric cancer metastasis. Recent research has shown that Bmi-1 plays essential roles in inducing EMT in head and neck squamous cell carcinoma [49], and Li et al. [50] found that Bmi-1 can regulate EMT to promote migration and invasion of breast cancer cells. In our study, we found that Bmi-1 can drive the EMT process by activating the PI3K/ AKT pathway in gastric cancer, and miR-128 can drive EMT by curbing the expression of Bmi-1. We also found that SNAIL, a transcriptional repressor, can directly regulate the expression of miR-128 in gastric cancer. In fact, SNAIL also acts as a mesenchymal marker, as it can induce EMT by repressing E-cadherin and activating several signaling pathways, such as the signal transducer and activator of transcription 3 (STAT3) and v-akt murine thymoma viral oncogene homolog (AKT) pathways [20, 51]. Here, our findings revealed a novel positive feedback loop. First, SNAIL bound directly to transcription factor binding sites of the human pri-miR-128 stem-loop E-box1 and E-box2, with resulting in down-regulation of miR-128 expression. Second, the expression of Bmi-1 was up-regulated in parallel with low expression of miR-128. Finally, increased Bmi-1 drove the EMT process via the PI3K/AKT pathway, and the expression of SNAIL was up-regulated. These findings reveal a novel cascade in gastric cancer oncogenic transformation.

In conclusion, our results showed that miR-128 is down-regulated in gastric cancer, and the down-regulation results from the methylation of SNAIL and miR-128 CpG island DNA. In addition, miR-128 can inhibit gastric cancer migration, invasion, proliferation, and metastasis by targeting Bmi-1 *in vivo* and *in vitro*. Thus, miR-128 is a potential biomarker for gastric cancer diagnosis and prognosis. Finally, miR-128 can inhibit epithelial-to-mesenchymal transition by targeting Bmi-1 and activating the PI3K/AKT pathway in gastric cancer cells. Appreciation of the SNAIL/miR-128 axis provides a novel insight into the mechanism of gastric cancer oncogenic transformation.

## MATERIALS AND METHODS

### Human tissue specimens and cell lines

This study utilized fresh tissues, including 135 human gastric cancer samples and adjacent normal mucosal tissues derived from 135 patients who underwent surgery at the Department of Surgery, Tongji Hospital of Tongji Medical College between 2011 and 2012. Each tissue was divided into two parts, one part was fixed in 10% neutral buffered formalin and embedded in paraffin, the other one was stored at −80°C for further processing.

This study was conducted according to the ‘Biomedical Research Involving Human Ethics Review (Tentative)’ regulations of the Ministry of Health and the Declaration of Helsinki on Ethical Principles for Medical Research Involving Human Subjects. All samples were obtained with the informed consent of the patients, and the experiments were approved by the Institutional Review Board of Tongji Hospital, Tongji Medical College, Huazhong University of Science and Technology. All participants provided written informed consent to participate in this study.

The SGC-7901, HGC-27, AGS, MKN-45 and N87 cell lines were obtained from the American Type Culture Collection (ATCC; Manassas, VA, USA), and the GES-1 cell line was purchased from the Type Culture Collection of the Chinese Academy of Sciences(Shanghai, China). The cell lines were cultured in RPMI1640 (HyClone, Logan Utah USA) supplemented with 10% fetal bovine serum (FBS) and were incubated at 37°C with 5% CO_2_.

### Primers, RNA isolation, and miRNA detection

The primers for miR-128 and U6 were produced using a miScript Primer Assay kit (Qiagen Dusseldorf Germany). The sequences of the miRNAs used in this study were as follows: miR-128, UCACAGUGAACC GGUCUCUUU and U6, CGCAAGGAUGACACGCAAAUUCGUGAAGCGUUCCAUAUUUUU. The reverse primers were also used in the reverse transcription step. Total miRNA was extracted from cultured cells and human tissue specimens using RNAiso for Small RNA (TaKaRa Bio, Otsu, Japan) according to the manufacturer's instructions. Poly-A tails were added to miR-128 and U6 with the miRNA Reaction Buffer Mix (TaKaRa Bio), and then, cDNA was synthesized from 5 ng of total RNA using a miRNA PrimeScript RT Enzyme Mix (TaKaRa Bio). Real-time PCR was performed in a CFX96™ Real-Time PCR Detection System (Bio-Rad) with SYBR® Premix Ex Taq™ II (TaKaRa Bio). The PCR conditions were 95°C for 30 s, followed by 40 cycles of 95°C for 5 s and 60°C for 30 s. The data were normalized against the U6 snRNA. After amplification, a melting curve analysis was performed to confirm the specificity of the products. Expression levels of the miRNAs were calculated by cycle threshold (Ct) values with SDS 2.0 software (Applied Biosystems). The concentrations from serum, tissues or cell line samples were normalized using the 2^−ΔΔCt^ method relative to U6 small nuclear RNA (RNU6B). The value of ΔCt was calculated by subtracting the Ct values of RNU6B from the Ct values of the miRNAs of interest in the study. The values of ΔΔCt were then calculated by subtracting the ΔCt of the control samples from the ΔCt of the cancer samples. The change in gene expression was calculated using the equation 2^−ΔΔCt^.

### DNA methylation analysis

Genomic DNA from gastric cancer cell lines and the GES-1 cell line was purified using DNAzol(Takara). Sodium bisulfite conversion was conducted using a Qiagen Epitect Bisulfite Kit (Qiagen) according to the manufacturer's instructions. The methylation states were determined by Methylation-specific PCR (MSP). The primers for methylated or unmethylated DNA were designed by the MethyPrimer tool (http://www.urogene.org/cgi-bin/methprimer/methprimer.cgi). The sequences of primers were as follows:the methylation forward primer,5′-TAGTAAAGCGAGAATTTCGC-3′ and the reverse primer, 5′-CTAACCGCCGAAAATAAAC-3′; the unmethylation forward primer, 5′-GTAGTAAAGTGAG AATTTTGT-3′and the reverse primer, 5′-ACTAACCAC CAAAAATAAAC-3′.

### Oligonucleotide transfection

miR-128 mimics and cont-miR were synthesized by Sangon Biotechnology (Sangon, Shanghai, China), and cotransfections were performed with Lipofectamine 2000 (Invitrogen). The oligonucleotides (GenePharma, Shanghai, China) were as follows: miR-128 mimics, 5′-UCACAGUGAACCGGUCUCUUU-3′(sense) and 5′-AGAGACCGGUUCACGGUGAUU-3′ (anti-sense); scrambled miRNA (NC), 5′-UUCUCCGAA CGUGUCACGUTT-3′(sense) and 5′-ACGUGACACGU UCGGAGAATT-3′ (anti-sense).

### pcDNA expression plasmids and RNA-interference

The ORF sequences of TWIST, SNAIL, ZEB1and Bmi-1 were amplified from genomic DNA isolated from the AGS cell line and were then subcloned into a GV230 vector(GeneChem Corporation, Shanghai, China). The plasmid was transfected into gastric cancer cells using Lipofectamine 2000 (Invitrogen). AGS cells that were stably transfected with Bmi-1 were selected using 2μg/μL puromycin (Invitrogen, Cergy-Pontoise, France) 48 h after the transfection.

SNAIL and Bmi-1 expression was silenced by transfecting cells with the following targeted siRNA sequences(Sangon Biotech, Shanghai, China) using Lipofectamine 2000 (Invitrogen): si-SNAIL sequences,(#1) 5′GCCUUCAACUGCAAAUACU and (#2) 5′AGUAUU UGCAGUUGAAGGC(where not specified, siRNA #1 was used); si-Bmi sequences,(#1)5′CCGACCACUA CUGAAUAUTTand (#2) 5′GGAUCGGAAAGUAAACA AATTand (#3) 5′CCAGAUUGAUGUCAUGUAUTT (where not specified, siRNA #1 was used); Control siRNA (siC) sequence,5′GATAGGTCATGACTGCCC'3.

### miRNA gene cloning and ectopic expression

The human miR-128 gene was PCR-amplified from normal genomic DNA and cloned into a lentiviral vector. The lentiviruses were generated by the cotransfection of HEK293T cells with plasmids pGC-LV, pHelper 1.0 and pHelper 2.0 using Lipofectamine 2000 (Invitrogen). Viruses were harvested 48 h post-transfection, and the infections were performed in the presence of 2 mg/mL polybrene (GeneChem Corporation). Control miRNA virus was purchased from GeneChem Corporation (Shanghai, China). AGS cells that were stably infected with miR-128 were selected using 2μg/μL puromycin (Invitrogen) 48 h after the infection.

### Luciferase reporter assay

We constructed a 3-kb fragment upstream of the human miR-128-2 stemloop, which contains2 conserved E-box motifs, 1 at -991 bp (E-box 1, CACATG) and the other at -26 bp (E-box 2, CACATG), and inserted the fragment into the luciferase reporter plasmid PsicheckTM-2(Sangon Biotech, Shanghai, China). Then, we cotransfected this plasmid together with different vectors expressing TWIST, SNAIL and ZEB1 into AGS cell lines. si-SNAIL was also cotransfected with the miR-128 stem-loop plasmid into AGS cell lines, and the luciferase activity was observed. A PsicheckTM-2 Dual-Luciferase miRNA target expression vector was used for 3′UTR luciferase assays (Sangon Biotech, Shanghai, China). The 3′UTR vector of BMI-1and TGFβR1containing an intact miR-128 recognition sequence was purchased from Geneseed Biotech Co. (Guangzhou, China). There is one binding site in the Bmi and TGFβR1 3′UTR, so we designed primer sequences for the mutant 3′UTR: Bmi-1,(forward)5′-CATTACTTTTACATATATTGCTGGCCCTTCTGCTTTC-3′ and (reverse)5′-GAAAGCAGAAGGGCCAGCAATATATGTAAAAGTAATG-3′; TGFβR1,(forward) 5′-CCTCAGAATAAGATCACAGTGATAAAAGGACTTC-3′ and (reverse)5′-GAAGTCCTTTTATC ACTGTGAT CTTATTCTGAGG-3′. For the luciferase assay, Lipofectamine 2000 was used to cotransfect AGS cells with the miR-128 and PsicheckTM-2 Dual-Luciferase miRNA target expression vectors containing wild-type or mutant target sequences. The firefly luciferase activity was measured using a Dual-Luciferase Assay (Promega, Madison, WI, USA) 18 h after transfection, and the results were normalized against Renilla luciferase. Each reporter plasmid was transfected at least three times (on different days), and each sample was assayed in triplicate.

### Chip assay

The ChIP assay was performed using a ChiP-IT enzymatic kit (Active Motif, Carlsbad, CA) following the manufacturer's instructions. After that, Snail was immunoprecipitated using specific antibodies (Santa Cruz Biotechnology CA, USA). The attached DNA was prepared using proteinase K and further purified using a phenol/chloroform procedure. PCR was performed using the following primer sets:E-box-1, (forward) 5′GCCTGGAACTGGAGGGTAAC'3 and (reverse) 5′GGGAACCCAGCCAAGAGATA'3; E-box-2, (forward) 5′CGTGTCTCGGTGGAACTCTG'3 and (reverse) 5′GTG GCTGCCGGGCTC'3. An amplicon at a distance of 2,116 base pairs upstream of the human miR-128-2 stem-loop was used as negative control: (forward) 5′GTCTTTTCTAAAGGAACAAAACGCT'3 and (reverse) 5′GCTCTGGCAAACTTTGGTGG'3.

### Antibodies and immunoblotting

Antibodies against fibronectin, DCX, E2F3, TGFBR1 and WEE1were purchased from Santa Cruz Biotechnology (CA, USA). phospho-Akt, SIRT1, FBXW7, SNAIL and vimentin were purchased from Abcam (Cambridge, UK), and total Akt, N-cadherin, E-cadherin, NTRK3, RELN, EGFR, GAPDH and BMI1were from BD Biosciences (USA). HRP-conjugated goat anti-rabbit IgG was purchased from Santa Cruz Biotechnology. Total protein was extracted from the transfected cells and gastric cancer tissues using RIPA lysis buffer (Beyotime, China) according to the manufacturer's instructions. After the whole-cell protein extracts were quantified using a BCA protein assay, equivalent amounts of cell lysates were resolved by 10% SDS polyacrylamide gel electrophoresis and were transferred onto a polyvinylidene fluoride membrane, which was then blocked in 5% non-fat milk in TBST for 1 h at 4°C. The blots were then incubated with primary antibodies. After incubation with HRP-conjugated secondary antibodies, the protein bands were visualized using an enhanced chemiluminescence reagent (Millipore, Billerica, MA, USA).

### Cell viability and colony formation assays

The transfected cells were seeded into 96-well plates at a density of 1×10^4^ cells/well. An MTT solution (20 ml of 5 mg/ml MTT) was added to the cultures (for a total volume of 200 ml) and incubated for 4 hat 37°C. Following the removal of the culture medium, the remaining crystals were dissolved in DMSO, and the absorbance at 570 nm was measured. For the colony formation assay, cells were seeded at a low density (1000 cells/plate) and allowed to grow until visible colonies appeared. The cells were then stained with Giemsa, and the colonies were counted.

### Migration and invasion assays

For the Transwell migration assays, 1×10^4^ cells were plated in the top chamber with a non-coated membrane (24-well insert; 8 μm pore size; BD Biosciences). For the invasion assays, 2×10^5^ cells were plated in the top chamber with a Matrigel-coated membrane (24-well insert; 8 mm pore size; BD Biosciences). For both assays, the cells were plated in a serum-free medium, and medium supplemented with 10% serum was used as a chemoattractant in the lower chamber. The cells were incubated for 16 h at 37°C and 5% CO_2_ in a tissue culture incubator. After 16 h, the non-migrated/non-invading cells were removed from the upper sides of the Transwell membrane filter inserts using cotton-tipped swabs. The migrated/invaded cells on the lower sides of the inserts were stained with Giemsa, and the cells were counted.

### Subcutaneous injection

Male athymic nude mice 6 to 8 weeks of age were obtained from the Animal Experimental Center of Tongji Hospital of Tongji Medical College and were acclimated for 2 weeks. This study was conducted in strict accordance with the recommendations of the Guide for the Care and Use of Laboratory Animals of Tongji Hospital of Tongji Medical College. The protocol was approved by the Committee on the Ethics of Animal Experiments of Tongji Hospital of Tongji Medical College. All surgeries were performed under sodium pentobarbital anesthesia, and all efforts were made to minimize suffering. Equal numbers of AGS cells (10^6^) with forced expression of miR-128 or cont-miR, with or without Bmi-1 restoration, were suspended in 100 μl of PBS and injected subcutaneously into the right rear flank of each mouse (5 mice per group). The mice were sacrificed 5 weeks later, and the tumors were divided into two parts. One part was fixed in formalin for subsequent immunohistochemical analysis, and the other part was preserved in liquidnitrogen for qRT-PCR.

### Tail vein injection

AGS cells (10^6^ cells in 200 ml of PBS) were injected directly into the lateral tail vein of 6- to 8-week-old male athymic nude mice. Each group consisted of 7 mice. Metastasis in mammary tumors, mice liver and lung were measured using a Fluorescence Imager (IVIS Spectrum, Caliper LifeSciences, Hopkinton, MA, USA) at 4 different time points (1, 3, 5 and 7 weeks after injection). After monitoring with the Imager, mice were sacrificed, and the lungs were fixed in 10% neutral buffered formalin and embedded in paraffin for histological examination.

### Immunohistochemistry

Tumors embedded in paraffin were sectioned onto positively charged microscope slides. The sections were deparaffinized in xylene, hydrated in graded alcohol, and pretreated for antigen retrieval in citrate buffer for 20 min in a 98°C steamer(Bmi-1 and Brdu). The sections were incubated at 4°C overnight with Brdu (1:200, Maixin, Fuzhou, China) and Bmi-1 (1:100BD Biosciences USA). Immunostaining was performed using an UltraSensitive S-P Detection Kit (KIT-9720, Maixin, Fuzhou, China), and the color was developed using a DAB kit (PW017, Sangon Biotech, Shanghai, China). A TUNEL assay was performed with an In Situ Cell Death Detection Kit (Roche) according to the manufacturer's instructions.

### Statistical analysis

SPSS 17.0 software was used for the statistical analysis. The data are presented as the means ± standard deviation (s.d.). Group comparisons were performed using Student's t-test. Receiver operating characteristic (ROC) curves and the area under the ROC curve (AUC) were used to assess the feasibility of tissue miRNA levels as a diagnostic tool for detecting gastric cancer. For disease progression, Kaplan-Meier (log-rank test) analysis was performed. Spearman's rank test was used to evaluate the relationships among the relative expression levels of miR-128 and Bmi-1 in gastric cancer tissues. Differences were considered significant if p < 0.05.
